# Treating Post-traumatic Stress Disorder in Patients with Multiple Sclerosis: A Randomized Controlled Trial Comparing the Efficacy of Eye Movement Desensitization and Reprocessing and Relaxation Therapy

**DOI:** 10.3389/fpsyg.2016.00526

**Published:** 2016-04-21

**Authors:** Sara Carletto, Martina Borghi, Gabriella Bertino, Francesco Oliva, Marco Cavallo, Arne Hofmann, Alessandro Zennaro, Simona Malucchi, Luca Ostacoli

**Affiliations:** ^1^Clinical Psychology and Psychosomatics Service, University Hospital San Luigi Gonzaga, University of TurinOrbassano, Italy; ^2^Clinical and Biological Sciences Department, University Hospital San Luigi Gonzaga, University of TurinOrbassano, Italy; ^3^Neurologia 2 – Centro di Riferimento Regionale Sclerosi Multipla, Azienda Ospedaliero-Universitaria San Luigi GonzagaOrbassano, Italy; ^4^eCampus UniversityNovedrate (CO,) Italy; ^5^Department of Mental Health, Azienda Sanitaria Locale Torino 3Turin, Italy; ^6^Facharzt für Psychosomatische und Innere Medizin, Eye Movement Desensitization and Reprocessing Institut DeutschlandBergisch Gladbach, Germany; ^7^Department of Psychology, University of TurinTurin, Italy

**Keywords:** multiple sclerosis, PTSD, EMDR, relaxation therapy, stress

## Abstract

**Objective:** Multiple Sclerosis (MS) is a demyelinating autoimmune disease that imposes a significant emotional burden with heavy psychosocial consequences. Several studies have investigated the association between MS and mental disorders such as depression and anxiety, and recently researchers have focused also on Post-traumatic Stress Disorder (PTSD). This is the first study that investigates the usefulness of proposing a treatment for PTSD to patients with MS.

**Methods**: A randomized controlled trial with patients with MS diagnosed with PTSD comparing Eye Movement Desensitization and Reprocessing (EMDR; *n* = 20) and Relaxation Therapy (RT; *n* = 22). The primary outcome measure was the proportion of participants that no longer meet PTSD diagnosis as measured with Clinician Administered PTSD Scale 6-months after the treatment.

**Results**: The majority of patients were able to overcome their PTSD diagnosis after only 10 therapy sessions. EMDR treatment appears to be more effective than RT in reducing the proportion of patients with MS suffering from PTSD. Both treatments are effective in reducing PTSD severity, anxiety and depression symptoms, and to improve Quality of Life.

**Conclusion**: Although our results can only be considered preliminary, this study suggests that it is essential that PTSD symptoms are detected and that brief and cost-effective interventions to reduce PTSD and associated psychological symptoms are offered to patients, in order to help them to reduce the psychological burden associated with their neurological condition.

**Trial registration**: NCT01743664, https://clinicaltrials.gov/ct2/show/NCT01743664

## Introduction

Multiple Sclerosis (MS) is a demyelinating autoimmune disease of the central nervous system that affects both the brain and the spinal cord by destroying the myelin sheath that protects the nerve fibers, leaving plaques or scars on the damaged sites. MS typically onsets during young age, and thus it poses a significant emotional burden with heavy psychosocial consequences. The disease substantially interferes with daily activities and family, social and working life, disturbs emotional well-being, and reduces Quality of Life (QoL) (Janssens et al., 2003).

Several studies have focused on the association between MS and psychiatric illnesses, such as depression (Siegert and Abernethy, 2005; Wallin et al., 2006; Beiske et al., 2008; Feinstein, 2011; Moore et al., 2012) and anxiety (Zorzon et al., 2001; José Sá, 2008), but only recently have researchers focused on Post-traumatic Stress Disorder (PTSD) associated to MS.

Relation between stress and MS relapses is complex and reciprocal. An increasing amount of research (Mohr et al., 2004; Mohr, 2007; Mitsonis et al., 2009) points toward an association between stressful life events and relapse of MS, proposing that stress may enhance the risk of exacerbation (Nisipeanu and Korczyn, 1993; Brown et al., 2006). Moreover recent studies focusing on psychoneuroimmunology are gaining importance, proposing that stress may influence immune function via the autonomic nervous system and the hypothalamus-pituitary adrenal axis (Kern and Ziemssen, 2008; Karagkouni et al., 2013).

Since chronic and potentially life-threatening illness was explicitly included as a stressor that could precipitate PTSD in DSM-IV (American Psychiatric Association, 1994), it was therefore possible for individuals who suffer from MS to receive a PTSD diagnosis related to their illness. From then on there was a spread of empirical studies on Post-traumatic symptoms in patients with different medical illnesses (Smith et al., 1999; Kangas et al., 2002; Tedstone and Tarrier, 2003; Einsle et al., 2012). As pointed out by some studies (Tedstone and Tarrier, 2003; Jackson et al., 2007), despite the fact that experience of a serious medical illness can be undoubtedly stressful, some aspects of this experience differ from what is traditionally defined and widely studied as a trauma, such as serious car accidents, sexual assaults, earthquakes or exposures to combat. At the same time stressful experiences such as the communication of the diagnosis, the information provided on the prognosis, some debilitating treatments or disabling symptoms seem to be potential traumatic events such as those already considered in the PTSD literature (Tedstone and Tarrier, 2003). The existential threat due to a life-threatening illness may be more comparable to long-term trauma, such as that experienced in abusive relationships. As in those cases, the traumatic experience that promotes PTSD does not derive from a single event (Tedstone and Tarrier, 2003; Chalfant et al., 2004), but from the cumulative effect of these negative experiences (Turner and Lloyd, 1995; Alonzo, 2000).

To date, only three studies have evaluated the prevalence and the characteristics of PTSD secondary to MS (Chalfant et al., 2004; Counsell et al., 2013; Ostacoli et al., 2013). All these studies included different clinical types of MS (Relapsing-Remitting, Primary Progressive and Secondary Progressive). The first study evaluated a small sample of MS patients and found a relatively high prevalence of PTSD (*n* = 9/58, i.e., 15.5%), similar to that found in studies on cancer patients (Andrykowski and Cordova, 1998; Jacobsen et al., 1998). Counsell et al. (2013) conducted an Internet survey and found that almost 55.1% of their sample indicated that having MS was at least somewhat traumatic. Ostacoli et al. (2013) found a significant lower prevalence (12/232; 5.2%) than previous studies, probably due to different tools being used for PTSD diagnosis.

Moreover, none of these studies found an association between PTSD and clinical indices of MS, such as duration, severity, clinical type of MS and degree of disability, confirming that PTSD is more related to individual factors than to the precipitating event (Brewin et al., 2000).

Several studies have highlight that PTSD, if not treated, is long-lasting (Van Etten and Taylor, 1998; Andrykowski et al., 2000; Peris et al., 2011) and it may be a highly debilitating and impairing condition with also huge societal costs (Kessler, 2000; Galatzer-Levy et al., 2013; Tuerk et al., 2013).

Although there is extensive evidence on the effectiveness of treatments for PTSD, to date no study has been conducted to investigate the usefulness of proposing a treatment for this disorder to patients with MS. Effective treatments for PTSD include individual Trauma Focused CBT (TF-CBT), Eye Movement Desensitization and Reprocessing (EMDR), and stress management/relaxation training (Silver et al., 1995; Taylor et al., 2003; Bisson and Andrew, 2007; Tol et al., 2013), but clinical guidelines indicate that trauma-focused therapies, such as EMDR and TF-CBT are more effective than non trauma-focused intervention (National Collaborating Centre for Mental Health (UK), 2005).

Both EMDR and Relaxation Therapy (RT) were effectively used with patients suffering from other medical diseases such as chronic pain (Grant and Threlfo, 2002; Schneider et al., 2008; Chang et al., 2015), fibromyalgia (Friedberg, 2004; Theadom et al., 2015), myocardial infarction (Arabia et al., 2011; Whalley et al., 2014) and cancer (Yoo et al., 2005; Capezzani et al., 2013).

The main purpose of this study was to investigate the efficacy of EMDR in treating MS-related PTSD as compared to RT, in order to prove the role of EMDR as an elective intervention also in this specific population. Furthermore, as a secondary aim, we evaluated the efficacy of EMDR and RT on Post-traumatic Stress-associated symptoms (i.e., anxiety and depression), and on QoL and fatigue.

Lastly, we aimed to detect possible differences between these two treatments (EMDR vs. RT) at the follow-up evaluation for Post-traumatic Stress-associated symptoms and QoL.

## Materials and Methods

This was a randomized controlled clinical trial where two active treatments (EMDR and RT) were compared with a restricted randomization in a 1:1 ratio, conducted in Italy. The trial registration number is NCT01743664.

### Participants

Patients with MS were consecutively recruited from 2010 to 2013 from the Regional Reference Centre for Multiple Sclerosis (CReSM) affiliated with the University Hospital San Luigi Gonzaga of Orbassano, Turin, Italy. This study was approved by the Research Ethics Committee of the University Hospital San Luigi Gonzaga. Informed consent was obtained from all the participants.

Inclusion criteria were as follows: (1) definite diagnosis of a relapsing-remitting and primary or secondary progressive MS disease (McDonald Criteria) (Polman et al., 2011); (2) age between 18 and 65 years; (3) clinically inactive phase of the disease; (4) fluent Italian speaker; (5) legal capacity to consent to the treatment; (6) diagnosis of PTSD; (7) Post-traumatic symptoms present for at least 3 months; (8) willingness to suspend all concomitant psychological treatment; (9) suspension of all psychotropic medications at least 1 month before the treatment or maintenance at baseline level throughout the study.

Exclusion criteria were as follows: (1) presence of severe psychiatric disorders such as psychosis or bipolar disorder; (2) presence of severe medical conditions other than MS, such as diabetes, strokes or traumatic brain injuries; (3) drug or alcohol abuse; (4) suicide attempts; (5) overt dementia; (6) corticosteroid treatment during the previous 30 days.

### Recruitment and Measures

Participants were recruited with a two-step screening: the Impact of Event Scale-Revised (IES-R) was administered to all patients corresponding to the neurological inclusion and exclusion criteria, specifying in the instructions that they consider only the illness as the traumatic event. The IES-R (Horowitz et al., 1979; Weiss and Marmar, 1997) is a 22- item self-report questionnaire consisting of three subscales (eight items relate to intrusions, eight items evaluate avoidance, and six items assess hyperarousal). The scale assesses subjective distress caused by traumatic events. A cut-off equal to or above 33, which is the cut-off most widely recognized in the literature (Creamer et al., 2003), is considered indicative of Post-traumatic stress symptoms.

Patients with scores equal to or above the cut-off of 33 were assessed with the PTSD module of Structured Clinical Interview for DSM-IV (SCID) (First, 1997) in order to confirm diagnosis of PTSD. Only PTSD primarily related to MS was considered. We also assessed the presence of previous trauma other than that related to MS, but they were not relevant to diagnose PTSD in this study.

Then patients with a confirmed diagnosis of PTSD were assessed with the Clinician-Administered PTSD Scale (CAPS) (Blake et al., 1995), a clinical semi-structured interview based on the DSM-IV-TR with B (intrusion), C (avoidance), and D (hyperarousal); to determine the presence of a symptom, we utilized the rule “1,2”: a frequency score of 1 (scale 0 = “none of the time” to 4 = “most or all of the time”) and an intensity score of 2 (scale 0 = “none” to 4 = “extreme”) is required for a particular symptom to meet criterion (Weathers et al., 2001). A severity score is calculated by adding together the frequency and intensity scores of subscales. Additional questions assess Criteria A, E, and F. CAPS is considered the gold standard in assessing PTSD (Foa and Tolin, 2000; Weathers et al., 2001).

The following psychological questionnaires were also administered:

(1) Hospital Anxiety and Depression Scale (HADS) was developed to identify caseness (possible and probable) of anxiety disorders and depression among patients in non-psychiatric hospital clinics. It is divided into an Anxiety subscale (HADS-A) and a Depression subscale (HADS-D) (Zigmond and Snaith, 1983; Bjelland et al., 2002). Each subscale contains seven items, which are evaluated by the patient on a four-point Likert scale (0–3), so that the possible scores range from 0 to 21 for both anxiety and depression. For each subscale, a result of 0–7 represents the normal condition; 8–10 identify mild cases; 11–15 moderate cases; and 16 or above indicates the presence of severe cases (Crawford et al., 2001; Quelhas and Costa, 2009). The tool has also been validated for use with patients with MS (Honarmand and Feinstein, 2009).
(1) Chicago Multiscale Depression Inventory (CMDI) is a 42-item, self-report questionnaire which was developed to assess depression in MS and other chronic diseases. The subscales assess mood (dysphoria), vegetative symptoms (physical malfunctioning) and evaluative symptoms (self-criticism). The mood subscale alone provides a more conservative indication of depression than total CMDI score (Solari et al., 2004).
(1) Functional Assessment of QoL in MS (FAMS) is a factorially derived self-report scale designed to assess six primary aspects of QOL of patients with MS: Mobility, Symptoms, Emotional Well-Being, General Contentment Thinking and Fatigue, and Family/Social Well- Being (Cella et al., 1996; Patti et al., 2007).
(1) Trauma Antecedent Questionnaire (TAQ) is a questionnaire to assess the trauma load due to previous traumas. The TAQ asks for the frequency (never, rarely, commonly) of experiences assigned to 11 domains (ranging from positive experiences such as competence and safety to negative experiences such as neglect, physical, emotional, sexual abuse, and witnessing trauma), separately assesses four developmental periods including early childhood (0–6), middle childhood, (7–12), adolescence (13–18), and adulthood (19+) (Garieballa et al., 2006).
(1) Fatigue Severity Scale (FSS) is a nine item one-dimensional questionnaire assessing the severity, frequency and impact of fatigue on daily life (Krupp et al., 1989). The cut-off of 36 is indicative of a severe fatigue (Flachenecker et al., 2002).

Lastly, patients received a score on the Expanded Disability Status Scale (EDSS) (Kurtzke, 1983, 2015) from their neurologist, to assess the level of disability.

### Assessment Points and Randomization

The psychological assessments were administered at pre-intervention (T0), at post-intervention (T1) that was about 12–15 weeks later, and at follow-up (T2) 6 months after the end of the treatment. T2 was considered as the main assessment point throughout all the analyses. Assessments were independent and blind to treatment.

The research protocol was then proposed to patients with PTSD who met the inclusion/exclusion criteria, with an explanation of the aims of the study, its relevance for MS patients and the possibility that they may be included, by random assignment, in the treatment or control group for the period of the study, with the same timing and assessment tools. If they agreed, they signed the informed consent and they were randomized to the experimental group (EMDR) or to the control group (RT), using a block-wise randomization sequence (block size of 10). The sequence was determined by an independent statistical consultant using the “Random Number Generators” function in SPSS version 14.0.

### Interventions

Treatments were independent and blinded to the clinical psychologists conducting the clinical assessments. All the participants, regardless of the type of treatment, received 10 individual 60-min-long treatment sessions conducted over 12–15 weeks, preceded by two sessions for history taking with particular attention to any stressful episodes in their life histories.

Eye Movement Desensitization and Reprocessing treatment was administered in accordance with Shapiro’s protocol for traumatic events (Shapiro, 2001). In the first session the patient is trained to stabilization techniques, such as the Safe Place. These imaginary exercises are used as a coping strategy to reduce distress, and the patient can also practice them at home as homework, or wherever and whenever they are needed. In the successive sessions the patient is induced to recall the traumatic images related to the illness, focusing on his/her worst negative thoughts, images or body sensations, thus provoking emotional disturbance, while the therapist uses external stimuli (usually eye movements, or tapping). Between each set of stimulation the patient reports thoughts, feelings or images that become the focus of the ongoing reprocessing. The residual distress becomes the focus of the next session of EMDR and the process continues until the distress evoked by the traumatic events, both past and future-oriented, is reduced, and negative cognitions are replaced by positive ones.

The three clinicians in the EMDR condition had more than 6 years’ experience in the liaison setting, working at the Clinical Psychology and Psychosomatics Service, Mental Health Department of the University Hospital San Luigi Gonzaga, with Level II EMDR training and a minimum of 3 years of experience in treating patients with PTSD. They received extensive training and supervision in the manualized protocol established for the study from a certified senior EMDR instructor.

Relaxation therapy included a series of relaxation techniques, including diaphragmatic breathing, progressive muscle relaxation, visualization, cue-controlled relaxation, and rapid relaxation (van Kessel et al., 2008). Relaxation treatment was performed by two psychotherapists working in the same facility of the EMDR intervention. The two clinicians in the Relaxation condition were at MSc level or higher, with a certified training in relaxation techniques and they had a minimum of 3 years’ experience treating patients with PTSD.

### Statistical Analysis

Data were processed and analyzed using IBM SPSS (Statistical Package for Social Sciences) version 19.0.

Both parametric and non-parametric tests were used, in accordance with Shapiro–Wilk as a test for normality. Baseline group differences were assessed using Student’s *t*-test or Mann–Whitney *U* test to compare the two groups for continuous measures and Fisher’s Exact Test for categorical measures.

Fisher’s Exact Test was used to evaluate the association between the treatment group (EMDR vs. RT) and the PTSD diagnosis at T1 and at T2. Cramer’s V was used to calculate the effect size for the primary outcome (proportion of participants that no longer meet PTSD diagnosis as measured with CAPS at T2).

GLM repeated measures multivariate ANOVA (RM-MANOVA) was used to analyze the main pre- and post-intervention effects and interactions both between and within EMDR and RT groups. Pairwise comparison between groups were made by simple contrast and are reported as means difference with the Sidak correction 95% Confidence Interval (95%CI) for multiple comparisons.

An intention to treat analysis was not possible due to patients’ refusal of following evaluations.

All tests were two-sided and a *p* < 0.05 was considered statistically significant throughout all analyses.

## Results

**FIGURE 1:**
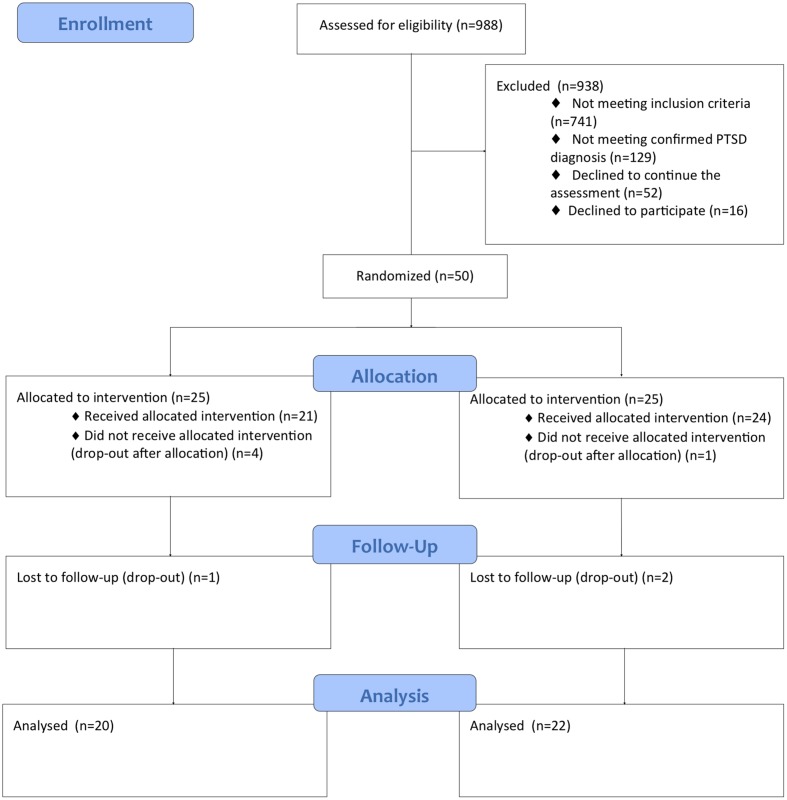
**Flow diagram of the progresss through the phases of the research**.

**Figure 1** shows the flow diagram with number of participants at each assessment stage.

A total of 988 patients with MS were screened with the IES-R; only 247 of these patients presented IES-R score above the cut-off (25%). Fifty-two patients (5.3%) refused to continue the evaluation (refusal rate: 21.1%). Of the other 195 patients, 66 meet the criteria of PTSD diagnosis (6.7%). Fourteen patients refused to participate (the reasons were mainly the distance of the place of residence from the place of treatment and the inability to independently reach the place of treatment) and two patients were excluded because they had a bipolar disorder.

Fifty patients were randomized: 25 were assigned to the EMDR intervention and 25 were assigned to the RT intervention.

Five patients did not begin the treatment (four in the EMDR group and one in the RT group) and three patients (one in the EMDR group and two in the RT group) attended only the first two sessions. These patients refused to continue with the assessment at T1 and T2 and therefore it was not possible to include them in the statistical analysis. Therefore a total of 42 patients (20 in the EMDR group and 22 in the RT group) completed the treatment and were assessed at the post-intervention (T1 and T2).

**Table 1** presents socio-demographic characteristics of these patients at baseline. There were no significant differences in demographics and in clinical characteristics between the two groups at baseline (T0).

**Table 1:** Demographic data of participants at baseline.

	EMDR (*N* = 20)	RT (*N* = 22)	*p*
	Mean (*SD*)/Median (IQR)	Mean (*SD*)/Median (IQR)	
Age (years)	39.52 (11.68)	40.66 (10.03)	0.736^a^
Education (years)	12.00 (7)	13.00 (4)	0.524^b^
Years since MS diagnosis	7.00 (10.50)	7.00 (10.25)	0.743^b^
Previous traumas	3.5 (6)	5 (7)	0.646^b^
EDSS	2.00 (4.50)	2.00 (1.60)	0.324^b^

	**n (%)**	**n (%)**	

Gender			0.445^c^
Female	15 (75)	19 (86.36)	
Male	5 (25)	3 (13.64)	
Employment status			0.321^c^
Unemployed	6 (30)	5 (22.73)	
Employed	8 (40)	14 (63.64)	
Pensioned	2 (10)	2 (9.09)	
Student	4 (20)	1 (4.54)	
Marital status			0.732^c^
Single	7 (35)	8 (36.36)	
Married	10 (50)	9 (40.91)	
Separated/divorced	1 (5)	3 (13.65)	
Widowed	0 (0)	1 (4.54)	
Cohabitee	2 (10)	1 (4.54)	
MS disease status			1.000^c^
Relapsing-remitting	17 (85)	19 (86.36)	
Primary progressive	1 (5)	1 (4.54)	
Secondary progressive	2 (10)	2 (9.1)	


*EMDR, Eye Movement Desensitization and Reprocessing group; RT, Relaxation Therapy group. ^a^Pearson’s independent samples t-test. ^b^Mann–Whitney *U* test. ^c^Fisher’s exact test.*

Firstly, we evaluated the proportion of patients that no longer meet PTSD diagnostic criteria at the end of the treatment (T1) and at follow-up assessment (T2). At T1 we found that 17 out of 20 patients in the EMDR group (85%) and 16 out of 22 patients (72.7%) in the RT group did not meet PTSD diagnosis criteria any longer. At T2, that is considered the primary outcome of our study, we found that all the patients in the EMDR group (20 out of 20, 100%) and 17 out of 22 patients (77.3%) in the RT group did not meet PTSD diagnosis criteria, with a statistically significant difference (χ^2^ = 5.160, *p* = 0.049; Cramer’s *V* = 0.350) in favor of EMDR.

Then we evaluated whether the different psychotherapy treatments (EMDR or RT) had a different impact on the psychological variables of interests. A Repeated-Measures MANOVA was performed on the pre- (T0) and post-intervention (T2) clinical scores (CAPS-Total, HADS-Anxiety, HADS-Depression, IES-R, FSS, CMDI-Mood, FAMS-Total), comparing group and time effects and interactions between group and time.

The RM-MANOVA yielded a significant pre-post main effect [*F*(7,34) = 45.244, *p* < 0.001; $\eta_{p}^{2}$ = 0.903], while no significant interaction was found between the pre-post measures and the treatment condition [*F*(7,34) = 0.631, *p* = 0.727; $\eta_{p}^{2}$ = 0.115]. Significant time effects were found across both groups on all variables, indicating that the mean participant scores improved from time 0 (pre-intervention) to time 1 (T2) in both groups without significant differences (**Table 2**).

**Table 2:** Comparison of clinical variables between pre- (T0) and post-treatment (T2) for the two groups (EMDR and RT).

	Pre-treatment		Post-treatment		*p^∗^*	$\eta_{p}^{2}$
				
	RT (*N = 22*)	EMDR (*N* = 20)	RT (*N* = 22)	EMDR (*N* = 20)		
CAPS-Total	44.41 (11.13)	44.55 (14.19)	19.54 (15.66)	16.60 (10.11)	<0.001	0.823
IES-R-Total	51.36 (9.58)	53.05 (12.87)	28.68 (19.39)	28.25 (18.28)	<0.001	0.596
HADS-Anxiety	11.32 (3.76)	12.10 (3.95)	7.64 (5.19)	7.40 (3.93)	<0.001	0.480
HADS-Depression	10.36 (4.09)	10.15 (3.38)	7.73 (4.73)	7.20 (3.93)	<0.001	0.295
CMDI-Mood	37.36 (11.00)	37.25 (12.36)	31.50 (13.55)	27.05 (13.16)	<0.001	0.280
FSS	43.95 (13.79)	43.10 (15.10)	39.18 (15.94)	37.60 (19.67)	0.029	0.114
FAMS-Total	96.73 (31.53)	88.80 (34.33)	109.82 (35.99)	102.75 (39.43)	0.001	0.232


*Data are mean (SD). RT, Relaxation Therapy group; EMDR, Eye Movement Desensitization and Reprocessing group. ^∗^significant pre–post effect, independent of the type of treatment (RT or EMDR).*

## Discussion

The present study is the first that evaluates the efficacy of two different types of psychological treatment for PTSD in patients with MS.

The most significant result emerging from this study is that the majority of patients were able to overcome their PTSD diagnosis after only 10 therapy sessions.

As expected, EMDR treatment appears to be more effective than RT in reducing the proportion of patients with MS suffering from PTSD. This is in accordance with guidelines that indicate that trauma-focused therapies, such as EMDR and TF-CBT, are more effective than non-trauma focused intervention, also having a more stable effect (National Collaborating Centre for Mental Health (UK), 2005; Bisson and Andrew, 2007; Bisson et al., 2013).

Both treatments were able to significantly reduce levels of PTSD symptoms and PTSD severity, showing that a non-trauma focused intervention such as RT could be as efficacious as EMDR in this specific population. As this is the first study aiming to investigate this important issue, it is possible that our sample does not allow possible differences between the two treatments to be revealed; future studies are needed to confirm these results.

Furthermore, both treatments are effective in reducing anxiety and depression symptoms even in a limited number of sessions, with an effect that lasts at least 6 months after the end of the psychological treatment. Both treatments worked on emotional stabilization and containment and maybe these components, that are present in both EMDR and RT, are the effective elements causing an improvement of anxiety and depression secondary to MS.

In line with previous studies focusing on EMDR and RT efficacy on QoL and fatigue related to other physical illness such as cancer or fibromyalgia (Friedberg, 2004; Yoo et al., 2005; Capezzani et al., 2013; Theadom et al., 2015), our study also showed QoL and fatigue improving significantly after both treatments. This result suggests that treatments that focus on Post-traumatic symptoms could also be useful to target lifestyle components, which are of great importance for patients who have to deal with a chronic disabling disease such as MS.

The results of this study could be interpreted also taking into account the recent changes in DSM-5, which states that medical disease *per se* can no longer be considered as a stressor event to qualify for a criterion A for PTSD diagnosis (American Psychiatric Association, 2013). DSM-5 also states that Adjustment Disorders are common accompaniments of medical illness and may be the major psychological response to a medical disorder (American Psychiatric Association, 2013). According to Kangas (2013), the stress reaction to a medical disease can include not only symptoms closely associated with PTSD but also a number of other symptoms that can be better understood by other diagnostic categories such as Adjustment Disorder. In fact, in this specific population the traumatic components seem to be part of a more complex adaptation process to the illness and the assessment of stress response symptoms may be complicated by the multiplicity and indeterminate nature of the stressor events and by the confounding effect of symptoms related to the illness or treatment (Kwakkenbos et al., 2014). Consistent with Kangas (2013), the results of this study suggest that EMDR could be a promising effective treatment also for Adjustment Disorder with Post-traumatic symptoms, in addition to PTSD.

Moreover, the majority of patients in our study reported previous traumas (e.g., sexual abuse, physical and psychological violence, complicated bereavement). This finding is in line with the accumulated burden of adversity model (Alonzo, 2000), according to which MS-related traumatic experiences (e.g., communication of the diagnosis, disabling treatments, possible exacerbation and/or recurrence of disease, functional impairment, fear of being confined to a wheelchair), when added to previous adverse experiences in life, may act as a trigger for developing PTSD or other trauma- and stressor-related disorders. Future studies should keep in mind these considerations and try to adapt already available treatments to the complex psychological and medical condition of these patients.

This study has also some strengths. It is the first ever study to evaluate the efficacy of psychological interventions for PTSD and associated symptoms in patients with MS.

The present study has some limitations. The number of included patients treated with EMDR and RT is not large, despite the very large group of patients screened. Another limitation is that there was no placebo or waiting list group, in order to control for the effect of time. This limit has an ethical implication, taking into account that PTSD symptom resolution does not occur with the passage of time, as shown in studies with cancer patients (Gonçalves et al., 2011). Also, depression in MS is largely chronic (Koch et al., 2015); therefore it’s extremely likely that untreated PTSD related to MS would not improve without any treatment.

Although our results can only be considered preliminary, this study suggests that EMDR is more effective than RT in reducing the proportion of patients with MS suffering from PTSD. Both EMDR and RT are effective for reducing Post-traumatic Stress symptoms and associated symptoms, also within a limited number of sessions.

These encouraging results suggest that it is essential that PTSD symptoms are detected and that brief and cost-effective interventions to reduce PTSD and associated psychological symptoms are offered to patients, in order to help them to reduce the psychological burden associated with their neurological condition.

## Author Contributions

LO, AZ, and AH were responsible for the conception and the design of the study. SC, MB, GB, FO, MC, and SM were responsible for data collection and analysis. SC wrote the article, which was critically revised by MB, GB, FO, MC, AH, AZ, SM, and LO. All authors read and approved the final version of the manuscript.

## Conflict of Interest Statement

AH is the president of EMDR Germany Institute. The other authors declare that there is no conflict of interest.
